# 15,16-Dihydrotanshinone I from the Functional Food *Salvia miltiorrhiza* Exhibits Anticancer Activity in Human HL-60 Leukemia Cells: *in Vitro* and *in Vivo* Studies

**DOI:** 10.3390/ijms160819387

**Published:** 2015-08-17

**Authors:** Jun-Jen Liu, Hsueh-Hsia Wu, Tzu-Ho Chen, Wan Leung, Yu-Chih Liang

**Affiliations:** 1 School of Medical Laboratory Science and Biotechnology, College of Medical Science and Technology, Taipei Medical University, No. 250 Wuxing St., Taipei 11031, Taiwan; E-Mails: jjliu_96@tmu.edu.tw (J.-J.L.); wuhh@tmu.edu.tw (H.-H.W.); dennisshit@hotmail.com (T.-H.C.); 2 Department of Radiology and Nuclear Medicine, Yuan’s General Hospital, No. 162 Cheng Kung 1st Road, Kaohsiung 80249, Taiwan; 3 Traditional Herbal Medicine Research Center, Taipei Medical University Hospital, No. 252 Wuxing St., Taipei 11031, Taiwan

**Keywords:** 15,16-dihydrotanshinone I, acute myeloid leukemia, c-Jun *N*-terminal kinase, Fas ligand, apoptosis

## Abstract

15,16-Dihydrotanshinone I (DHTS) is extracted from *Salvia miltiorrhiza* Bunge which is a functional food in Asia. In this study, we investigated the apoptotic effect of DHTS on the human acute myeloid leukemia (AML) type III HL-60 cell line. We found that treatment with 1.5 μg/mL DHTS increased proapoptotic Bax and Bad protein expressions and activated caspases-3, -8, and -9, thus leading to poly ADP ribose polymerase (PARP) cleavage and resulting in cell apoptosis. DHTS induced sustained c-Jun N-terminal kinase (JNK) phosphorylation and Fas ligand (FasL) expression. The anti-Fas blocking antibody reversed the DHTS-induced cell death, and the JNK-specific inhibitor, SP600125, inhibited DHTS-induced caspase-3, -8, -9, and PARP cleavage. In a xenograft nude mice model, 25 mg/kg DHTS showed a great effect in attenuating HL-60 tumor growth. Taken together, these results suggest that DHTS can induce HL-60 cell apoptosis *in vitro* and inhibit HL-60 cell growth *in vivo*; the underlying mechanisms might be mediated through activation of the JNK and FasL signal pathways.

## 1. Introduction

15,16-Dihydrotanshinone I (DHTS) is extracted from the *Salvia miltiorrhiza* Bunge (Tanshen), which is used as a dietary supplement or as an ingredient in functional foods in Asian countries. Recent reports demonstrated that Tanshen have many biological functions, such as treating cardiovascular diseases, especially angina pectoris and myocardial infarction [1,2]. Studies by us and others found that extracts of Tanshen exhibit significant antitumor activity through different mechanisms in various types of tumor cells. Among the compounds of Tanshen, DHTS has the strongest inhibitory activity against breast cancer cells through inducing G_1_-phase arrest and increasing loss of the mitochondrial membrane potential and cytochrome c release [3]. DHTS also significantly induced apoptosis in colorectal cancer cells, and ATF-3 might be involved in inducing apoptosis [4]. In addition, DHTS can induce apoptosis of prostate carcinoma cells via induction of endoplasmic reticular stress and/or inhibition of proteasome activity [5], and may have therapeutic potential for prostate cancer patients. In human hepatoma cells, DHTS also induced cell apoptosis through inducing reactive oxygen species (ROS) and the p38 pathway [6]. Tanshinone I, a compound of Tanshen, was shown to induce cancer cell apoptosis in human myeloid leukemia cells [7] and human non-small cell lung cancer [8], whereas another of Tanshen’s compounds, tanshinone IIA, also induced apoptosis in human HeLa [9] and rat glioma cells [10].

At present, two major apoptotic pathways have been addressed, including the intrinsic mitochondrial pathway and extrinsic death receptor pathway [11,12]. The mitochondrial membrane plays a crucial role in initiating the intrinsic apoptosis pathway, which can occur by decreasing antiapoptotic Bcl-2 family proteins, such as Bcl-2 and Bcl-x_L_, and increasing proapoptotic Bcl-2 family proteins, such as Bad and Bax, with various apoptotic stimuli. Overall, a decrease in the antiapoptotic protein/proapoptotic protein ratio results in cytochrome c release into the cytosol and causes pro-caspase-9 cleavage. On the other hand, the extrinsic apoptotic pathway is activated by various death receptors, such as Fas, and finally induces pro-caspase-8 cleavage. Cleaved caspase-8 can cleave Bid into truncated (t)Bid which interacts with Bax or Bak to cause cytochrome c release from mitochondria. Cleaved caspase-9 and -8 can subsequently activate downstream effector caspases, including caspase-3, which destroys the cellular machinery and leads to eventual cell death [13].

## 2. Results

### 2.1. 15,16-Dihydrotanshinone I (DHTS) Inhibited Cell Proliferation and Triggered Apoptosis

**Figure 1:**
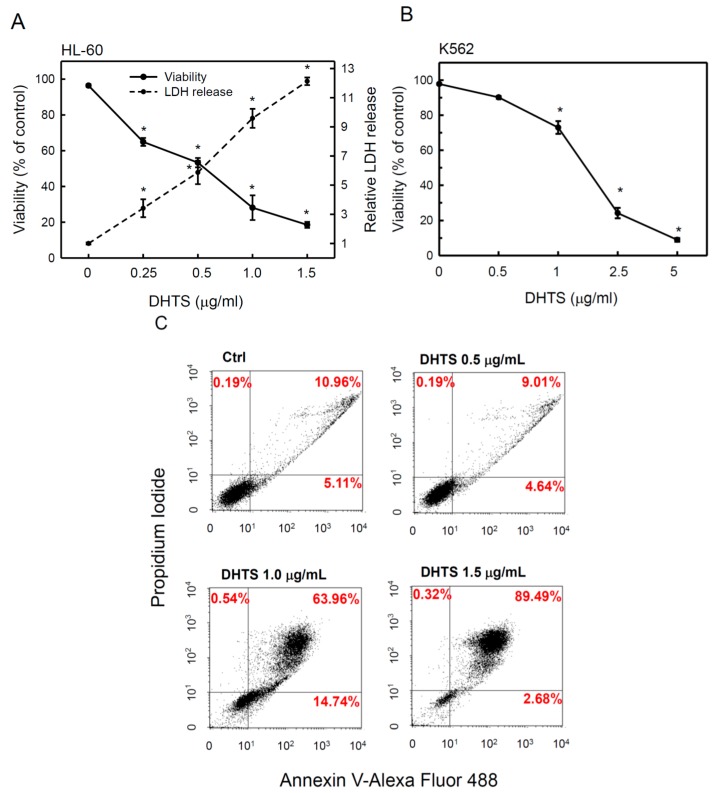
Effects of 15,16-dihydrotanshinone I (DHTS) on the cell viability and cytotoxicity of human HL-60 and K562 leukemia cells. (**A**) HL-60 cells were treated with various concentrations of DHTS for 24 h. Cell numbers and cytotoxicity were measured by counting viable cells using an MTT assay and lactate dehydrogenase (LDH) release, respectively; (**B**) K562 cells were treated with various concentrations of DHTS for 24 h. Cell numbers was measured by counting viable cells using an MTT assay; and (**C**) Cells were treated with various concentrations of DHTS for 24 h, and apoptotic cells were determined by FACS using the Annexin V-Alexa Fluor488 Apoptosis Assay Kit. Data are expressed as the mean ± S.D. of three independent experiments. * *p* < 0.05, compared to the control.

To examine whether DHTS can inhibit cell proliferation in a human hematopoietic malignancy, we used human promyelocytic leukemia HL-60 cells in all experiments of this study. HL-60 cells (5 × 10^4^ cells/mL) were cultured in RPMI medium containing 10% FBS and treated with various concentrations of DHTS for 24 h. Viable cells were determined by an MTT assay, and the results showed that DHTS dose-dependently inhibited cell proliferation in HL-60 cells with a 50% inhibitory concentration (IC_50_) of about 0.51 μg/mL (Figure 1A). Lactate dehydrogenase (LDH) is a stable cytosolic enzyme that is released upon cell lysis. Next, we determined the cytotoxicity of DHTS toward HL-60 cells by measuring the released LDH in culture supernatants. As shown in Figure 1A, DHTS treatment significantly increased the LDH release in a dose-dependent manner, indicating that DHTS caused significant cell death of HL-60 cells. In addition, DHTS also significantly inhibited proliferation of another human K562 chronic myelogenous leukemia cells, but it was less effective in K562 cells than that in HL-60 cells (Figure 1B).

Next, we examined whether DHTS-caused cell death was accompanied by an induction of apoptosis in HL-60 cells. HL-60 cells were treated with 0.5, 1.0 or 1.5 μg/mL of DHTS for 24 h and stained with PI and Annexin V-Alexa Fluor 488 followed by quantification of apoptotic cells by flow cytometry. Cells stained with Annexin V (Alexa Fluor 488 dye) represent early apoptotic cells and are shown in the lower right quadrant of the FACS histogram, and those stained with both Annexin V and propidium iodide (PI) represent late apoptotic cells and are shown in the upper right quadrant of the FACS histogram. As shown in Figure 1C, the late apoptotic cell population increased from 10.96% to 89.49% in HL-60 cells treated with 1.5 μg/mL DHTS. However, the early apoptotic cell population only increased in cells treated with 1.0 μg/mL DHTS. These results suggest that DHTS can suppress cell proliferation through inducing apoptosis in HL-60 cells.

### 2.2. DHTS Induced Apoptosis through Increased Bad/Bax Expression and Activation of Caspases

**Figure 2:**
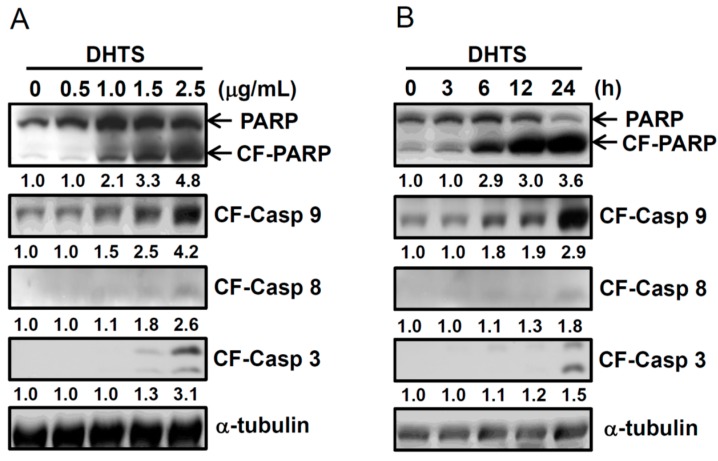
Effects of 15,16-dihydrotanshinone I (DHTS) on activation of caspases in human HL-60 promyelocytic leukemia cells. Cells were treated with (**A**) various concentrations of DHTS for 24 h or (**B**) 1.5 μg/mL DHTS for different time periods, and total cellular proteins were collected to determine protein expressions by Western blotting. CF, cleaved form; CF-casp-3, cleaved form of caspase-3; CF-casp-8, cleaved form of caspase-8; CF-casp-9, cleaved form of caspase-9. Relative intensities of CF-PARP and CF-caspases were quantified using ImageJ, normalized *versus* internal controls α-tubulin, and shown below the band pictures. Three independent experiments were performed and representative results are shown.

It is known that intrinsic and extrinsic apoptotic pathways can activate initiator caspase-8 and caspase-9, respectively, which then results in activation of effector caspase-3 and poly ADP ribose polymerase (PARP) cleavage by active caspase-3. To investigate whether intrinsic and/or extrinsic apoptotic pathways are involved in DHTS-induced apoptosis, we examined the expressions of cleaved caspase-8, cleaved caspase-9, cleaved caspase-3, and PAPR by Western blotting. HL-60 cells were treated with 0.5~2.5 μg/mL DHTS for 24 h, or 1.5 μg/mL DHTS for different time periods. As shown in Figure 2, DHTS significantly increased expressions of cleaved caspase-3, -8, and -9, and cleaved PARP in dose- and time-dependent manners in HL-60 cells.

**Figure 3:**
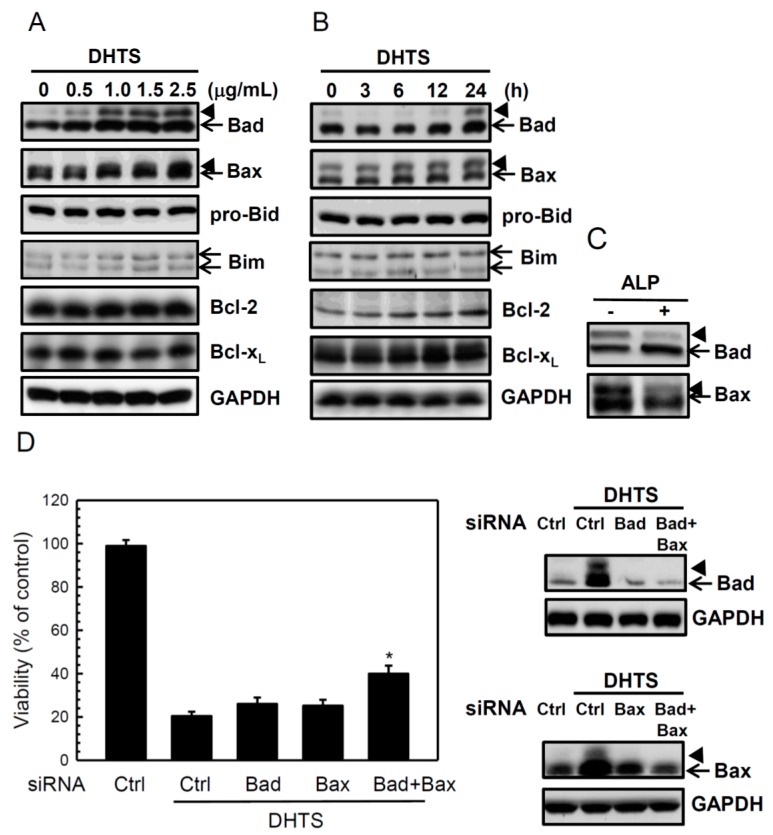
Effects of 15,16-dihydrotanshinone I (DHTS) on Bad and Bax expressions in human HL-60 promyelocytic leukemia cells. Cells were treated with (**A**) various concentrations of DHTS for 24 h or (**B**) 1.5 μg/mL DHTS for different time periods, and total cellular proteins were collected to determine protein expressions by Western blotting; (**C**) Total cellular lysate were collected from the cells treated with 1.5 μg/mL DHTS for 24 h, and incubated with or without alkaline phosphatase (ALP) in tube, and then determined protein expressions by Western blotting; and (**D**) Cells were transfected with Bad siRNA and/or Bax siRNAs by electroporation and then treated with 1.0 μg/mL DHTS for 24 h. Cell numbers was measured by counting viable cells using an MTT assay (**left** **panel**), and total cell lysates were used to detect the Bad and Bax protein levels by Western blotting (**right panel**). Arrowhead, a shifted band of Bad or Bax. Data are expressed as the mean ± S.D. of three independent experiments. * *p* < 0.05, compared to the column 2.

Bcl-2 family members are major regulators of apoptosis, such as the proapoptotic Bad, Bax, Bid, and Bim, and the antiapoptotic Bcl-2 and Bcl-x_L_. We found that DHTS dramatically increased proapoptotic Bad and Bax expressions and slightly increased Bim expression, but did not significantly change the expressions of Bid, Bcl-2, or Bcl-x_L_ (Figure 3A,B). Interestingly, a shifted band of Bad and Bax (Figure 3, arrowhead) appeared in the Western blot of DHTS-treated cells and could be decreased when they incubated with alkaline phosphatase (Figure 3C), suggesting that DHTS might induce phosphorylation of Bad and Bax in HL-60 cells. To confirm the roles of Bad and Bax on the DHTS-induced apoptosis, we used specific siRNAs to knockdown Bad and Bax in HL-60 cells. As shown in Figure 3D, neither Bad siRNA nor Bax siRNA could reverse the DHTS-induced cell death, however being combined with Bad siRNA and Bax siRNA significantly increased the cell viability. These results suggest that DHTS might induce apoptosis through both intrinsic and extrinsic apoptotic pathways.

### 2.3. Both FasL and c-Jun N-Terminal Kinase (JNK) Contributed to DHTS-Induced Apoptosis

**Figure 4:**
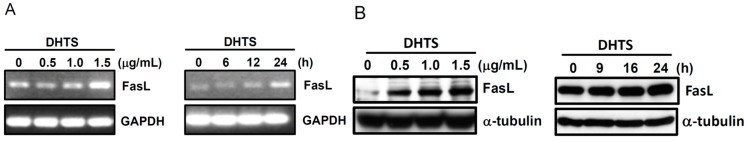

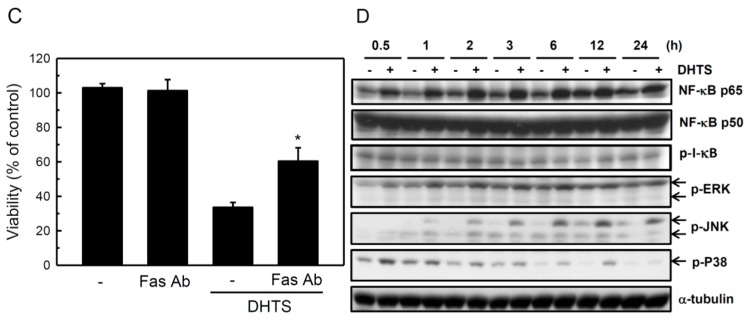
Effects of 15,16-dihydrotanshinone I (DHTS) on FasL and nuclear factor (NF)-κB expressions and mitogen-activated protein kinase (MAPK) phosphorylation in human HL-60 promyelocytic leukemia cells. (**A**,**B**) Cells were treated with various concentrations of DHTS for 24 h or 1.5 μg/mL DHTS for different time periods. (**A**) Total RNA was collected to determine FasL mRNA expression by an RT-PCR; (**B**) Total cellular proteins were collected to determine FasL protein expression by Western blotting; (**C**) Cells were treated with 1.0 μg/mL DHTS and 10 μg/mL anti-Fas blocking antibody for 24 h. Viable cell numbers were measured by an MTT assay. Data are expressed as the mean ± S.D. of three independent experiments. * *p* < 0.05, compared to the column 3; and (**D**) Cells were treated with 1.5 μg/mL DHTS for different time periods. Total cellular proteins were collected to determine protein expressions by Western blotting. p-ERK, phosphorylated extracellular signal-regulated kinase; p-JNK, phosphorylated c-Jun N-terminal kinase; p-p38, phosphorylated p38.

Since caspase-8 is activated by DHTS treatment, we next examine whether DHTS increased expression of the caspase-8 upstream signal molecule, FasL. HL-60 cells were treated with 0.5~1.5 μg/mL DHTS for 24 h or 1.5 μg/mL DHTS for different time periods. As shown in Figure 4A,B, DHTS induced FasL mRNA expression and also FasL protein expression, indicating that DHTS increased the FasL expression at the transcriptional level. To investigate whether FasL involved in DHTS-induced cell death, we used the anti-Fas blocking antibody to block the Fas/FasL interactions. The results indicated that anti-Fas blocking antibody significantly reversed the cell death in DHTS-treated cells, suggesting FasL was involved in DHTS-induced apoptosis in HL-60 cells (Figure 4C). NF-κB is a heterodimer or homodimer composed of NF-κB p50 and NF-κB p65 (or RelA), and NF-κB activation by phosphorylation and degradation of I-κB are linked to the induction of *FasL* gene expression. To examine whether DHTS-induced FasL expression is mediated through activation of NF-κB, we detected the expression of phosphorylated I-κB, NF-κB p65, and NF-κB p50 in HL-60 cells treated with DHTS for different time periods. As shown in Figure 4C, the expression of phosphorylated I-κB did not change; however, NF-κB p65 significantly increased in DHTS-treated cells. Moreover, c-Jun N-terminal kinases (JNKs) are members of MAPKs, and sustained phosphorylation of JNK was found to be associated with cell death in many reports. JNK phosphorylation occurred at 1 h and persisted to 24 h after DHTS treatment (Figure 4D). Moreover, phosphorylation of the other two MAPK members, ERK and p38, also increased in DHTS-treated cells in a time-dependent manner.

**Figure 5:**
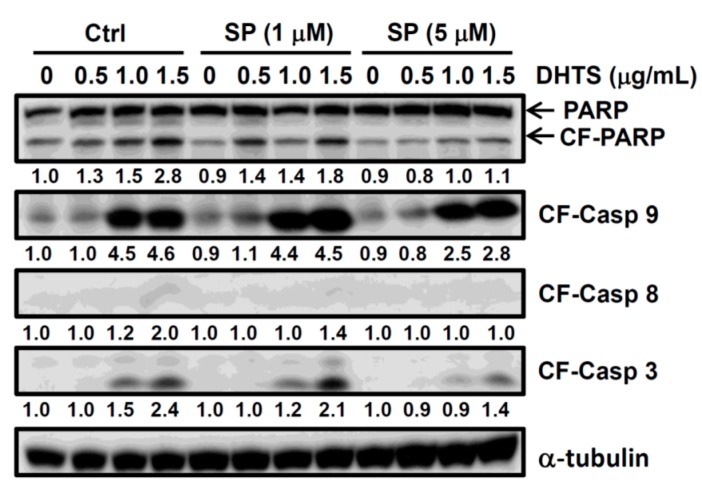
Effects of a c-Jun N-terminal kinase (JNK) inhibitor on 15,16-dihydrotanshinone I (DHTS)-induced cleavages of poly ADP ribose polymerase (PAPR) and caspases in human HL-60 promyelocytic leukemia cells. Cells were pretreated with the JNK inhibitor, SP600125, for 1 h, and treated with various concentrations of DHTS for 24 h. Total cellular proteins were collected to determine protein expressions by Western blotting. SP, SP600125; CF, cleaved form; CF-casp-3, cleaved form of caspase-3; CF-casp-8, cleaved form of caspase-8; CF-casp-9, cleaved form of caspase-9. Relative intensities of CF-PARP and CF-caspases were quantified using ImageJ, normalized *versus* internal controls α-tubulin, and shown below the band pictures. Three independent experiments were performed and representative results are shown.

To further confirm the role of JNK in apoptosis, cells were treated with the JNK inhibitor, SP600125, and expressions of cleaved caspases and PARP were detected. As shown in Figure 5, 5 μM SP600125 reversed increases in cleaved caspase-3, -8, and -9, and PARP in DHTS-treated cells. These results suggest that DHTS-induced apoptosis might be mediated through increases in FasL expression and JNK activation in HL-60 cells.

### 2.4. DHTS Inhibited Leukemia Tumor Growth in Nude Mice

**Figure 6:**
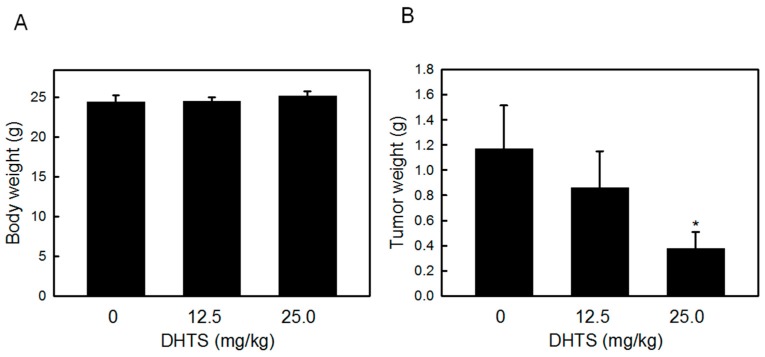
Effects of 15,16-dihydrotanshinone I (DHTS) on HL-60 tumor xenografts in nude mice. HL-60 cells were subcutaneously injected between the scapulas of athymic nude mice, and the mice received an i.p. injection of 12.5 or 25 mg/kg DHTS a day for a week. (**A**) Body weight and (**B**) tumor weight were measured at the end of the experiment. Values were obtained in five samples and are presented as the mean ± S.D. * *p* < 0.05 *versus* the control.

To examine the antitumor effects of DHTS on leukemia cells *in vivo*, we used a nude mice xenograft model. Athymic mice bearing HL-60 tumors were treated with 12.5 and 25.0 mg/kg DHTS once a day for a week. At the end of the experiment, the body weight and tumor weight were measured. As shown in Figure 6, 25 mg/kg of DHTS significantly inhibited tumor growth by about 68.0% compared to control tumors, and body weights remained unchanged between control and drug-treated mice. These results provide further evidence that DHTS might have significant applications for cancer therapeutic purposes.

## 3. Discussion

We herein showed that DHTS significantly induced apoptosis of human HL-60 leukemia cells at very low concentrations, as evidenced by a decrease in viable cell numbers and an increase in PI/annexin V-positive cells. We found that the intrinsic initiator, caspase-9, and the extrinsic initiator, caspase-8, were activated in DHTS-treated cells. DHTS also induced mRNA and protein expressions of FasL and sustained JNK phosphorylation. Blocking FasL/Fas interactions by anti-Fas blocking antibody could reverse the DHTS-induced cell death, and pretreatment with the JNK inhibitor, SP600125, limited the cleavage of caspase-3, -8, and -9, and PARP. Finally, we used an animal model to verify the antitumor potential of DHTS. These results suggest that DHTS induce apoptosis of HL-60 leukemia cells and inhibit tumor growth *in vivo*, and the underlying mechanisms might be mediated through both intrinsic and extrinsic apoptotic pathways.

Bcl-2 family proteins can be divided into three categories according to the BCL-2 homology (BH) domains, including multi-domain antiapoptotic proteins, multi-domain proapoptotic proteins, and BH3-only proapoptotic proteins [14]. BH3-only proapoptotic proteins, such as Bad, are cell death initiators that promote cell death by displacing Bax from binding to Bcl-2 and Bcl-x_L_. Evidence indicates that Bad is a latent killer until activated through transcriptional or post-translational mechanisms. The most common post-translational modification is obviously phosphorylation [15]. Phosphorylation of multiple serine residues was identified in Bad. Most findings demonstrated that Bad phosphorylation events inactivate its proapoptotic function; however, Bad phosphorylation at Ser 128 by Cdc2 limits it binding to 14-3-3 and therefore enhances the proapoptotic function of Bad in rat neuron cells [16]. Bax is a key component for inducing the intrinsic apoptosis pathway. Active Bax oligomers cause the release of cytochrome c from mitochondria and activate caspase-9 by cytosolic cytochrome c. In mammalian cells, phosphorylation of Ser163 and Ser184 of Bax may also participate in regulating Bax activity [17]. Phosphorylation of Ser184 by nicotine-activated Akt led to inhibition of Bax-dependent apoptosis [18]; however, dephosphorylation of Ser184 by PP2A resulted in Bax activation [19]. Akt is a key signal molecule for cell survival and proliferation, and it can phosphorylate and inactivate GSK3β. Another study showed that activated GSK3β phosphorylated Bax at Ser163 and then induced activation of Bax [20]. In this study, shifted Bad and Bax bands were found in Western blots of DHTS-treated cells (Figure 3A,B, arrowhead) and could be removed from the phosphate group by alkaline phosphatase (Figure 3C). They seem to be phosphorylated Bad and phosphorylated Bax; however more experiments are needed to fully understand which residues of Bad and Bax are phosphorylated and the actual roles of phosphorylated Bad and Bax on the DHTS-induced apoptosis. Bid is a BH3-only proapoptotic protein that is associated with extrinsic apoptosis pathways. FasL binding to the Fas receptor results in activation of the proapoptotic caspase-8 pathway by direct recruitment of FAS-associated proteins with a death-domain (FADD) [21]. Activated caspase-8 both leads directly into caspase-3 activation and cleaves Bid to generate truncated (t)Bid [22]. tBid helps the oligomerization of Bax into the outer mitochondrial membrane and stimulates the release of cytochrome c from mitochondria. Free cytochrome c finally activates caspase-3 by mediation through Apaf-1 and caspase-9. Our results found that DHTS activated caspase-8 (Figure 2) but did not change pro-Bid expression (Figure 3), suggesting that caspase-8 activated downstream caspase-3, thus bypassing the tBid/cytochrome c pathway.

Previous studies demonstrated that sustained JNK activation plays an important role in anticancer drug-induced apoptosis [23]. Activation of the JNK/c-Jun pathway can induce FasL expression in cisplatin-induced apoptosis [24]. JNK is able to induce FasL expression mediated by production of reactive oxygen species (ROS) in Epstein-Barr virus (EBV)-transformed B cells [25]. On the other hand, JNK was found to activate proapoptotic Bad, Bim, and Bax through different mechanisms. For example, JNK can phosphorylate 14-3-3, which results in Bad release from 14-3-3 [26]. In addition, JNK directly phosphorylates and inactivates the antiapoptotic Bcl-2 and Bcl-x_L_, thereby activating Bim and Bax [27]. In this study, we found that DHTS treatment induced JNK phosphorylation for long periods of time and FasL expression by HL-60 cells (Figure 4). However, the JNK-specific inhibitor, SP600125, and the ROS scavenger, *N*-acetyl cysteine (NAC), did not block FasL expression in DHTS-treated cells (data not shown). Additional experiments demonstrated that SP600125 could decrease the cleavage of PARP, and caspases-3, -8, and -9 (Figure 5), suggesting that DHTS-activated JNK might contribute to apoptosis through inhibiting antiapoptotic proteins and activating proapoptotic proteins.

The most common form of NF-κB is the p65/p50 heterodimer, which is known to participate in many physiological functions, such as immunity, inflammation, cell proliferation, and survival [28]. NF-κB is sequestered in the cytoplasm by binding with I-κB and is activated by a cascade of events leading to the phosphorylation of I-κB and subsequent degradation of I-κB. Free NF-κB is therefore translocated to nuclei and induces gene transcription through binding with *cis*-acting κB elements [29]. The *FasL* gene promoter contains several κB elements and can be induced by activated NF-κB [30]. In this study, we found that DHTS did not increase I-κB phosphorylation but significantly increased NF-κB p65 protein expression (Figure 4D), suggesting that DHTS-induced FasL expression might be mediated through the NF-κB pathway.

Previously, our studies have found that DHTS could induce apoptosis through intrinsic mitochondrial apoptotic pathway in breast cancer cells. Here, we further demonstrate that not only the mitochondrial pathway proteins, Bad and Bax, but also the extrinsic death receptor pathway, FasL were involved in DHTS-induced apoptosis in HL-60 leukemia cells. Upregulation of FasL expression by DHTS might be mediated through increasing JNK phosphorylation. Increase of both FasL as well as Bad and Bax results in activation of caspase cascade that finally contributes to cell apoptosis.

## 4. Materials and Methods

### 4.1. Materials

DHTS (PubChem CID: 11425923) was purchased from Xi’an Honson Biotechnology (Xi’an, China), and the purity was >90% according to a high-performance liquid chromatographic analysis. A stock solution of DHTS was prepared in dimethyl sulfoxide (DMSO) and stored at −20 °C. Anti-Bad, anti-Bcl-2, anti-Bcl-x_L_, anti-caspase-3, anti-caspase-8, anti-caspase-9, anti-PARP, anti-phospho-JNK, and anti-phospho-p38 antibodies were purchased from Cell Signaling Technology (Danvers, MA, USA); anti-NF-κB p50, anti-NF-κB p65, anti-p-I-κB, and anti-Bim antibodies were purchased Santa Cruz Biotechnology (Santa Cruz, CA, USA); anti-phospho-ERK and anti-FasL antibodies were purchased from BD Biosciences Taiwan (Taipei, Taiwan); anti-Bax and anti-GAPDH antibodies were purchased from GeneTex International (Hsinchu City, Taiwan); anti-Fas blocking antibody was purchased from MBL International (Woburn, MA, USA); the anti-α-tubulin antibody was purchased from Zymed Laboratories (South San Francisco, CA, USA).

### 4.2. Cell Culture and Transient Transfection

The human promyelocytic leukemia cell line, HL-60 (BCRC 60027), and human chronic myelogenous leukemia cell line, K562 (BCRC 60007), were obtained from the Food Industry Research and Development Institute (Hsinchu, Taiwan) and cultured in RPMI medium containing 10% heat-inactivated fetal bovine serum (FBS; Biological Industries, Kibbutz Beit Haemek, Israel). For knockdown of Bad and/or Bax, HL-60 cells (1.0 × 10^7^ cells) were transiently transfected with 200 nM of Bad siRNA and/or Bax siRNA (Santa Cruz) by electroporation at 250 V and 500 μF and then seeded in a 24-well plate for treatment with DHTS.

### 4.3. 3-(4,5-Dimethylthiazol-2-yl)-2,5-diphenyl Tetrazolium Bromide (MTT) Assay and Lactate Dehydrogenase (LDH) Release Assay

HL-60 cells or K562 cells (5 × 10^4^ cells) were seeded in a 24-well plate for 24 h and then treated with DHTS for another 24 h. The cell suspension was collected and centrifuged at 700× *g* for 1 min, and the cell pellet and supernatant medium were respectively used to determine viable cells and LDH release. To determine viable cells, the cell pellet was supplemented with 200 μL of fresh medium and 50 μL MTT (2 mg/mL) at 37 °C for 2 h in the dark. The medium was removed, and 200 μL DMSO and 25 μL Sorensen’s glycine buffer were added. The supernatant (100 μL) was put into a 96-well plate, and the absorbance at OD 570 nm was measured by an enzyme-linked immunosorbent assay (ELISA, HIDEX OY, Turku, Finland) plate reader. To determine LDH release, 50 μL of the above supernatant medium was added to a 96-well plate, and LDH activity was detected by a CytoTox 96^®^ Non-Radioactive Cytotoxicity Assay kit [31] (Promega, Madison, WI, USA) according to the manufacturer’s instructions.

### 4.4. Western Blot Analysis

Equal amounts of total cellular protein (50 μg) were resolved by 10% sodium dodecylsulfate (SDS)-polyacrylamide gel electrophoresis (PAGE) and transferred onto a polyvinylidene difluoride membrane (Millipore, Bedford, MA, USA) as described previously [32]. The membrane was then incubated with various primary antibodies and subsequently incubated with an anti-mouse or anti-rabbit immunoglobulin G (IgG) antibody conjugated to horseradish peroxidase (Santa Cruz Biotechnology) and visualized using enhanced chemiluminescence kits (Amersham, Arlington, IL, USA). For removal of phosphate groups from proteins, total cellular protein were collected in Gold lysis buffer [32] without phosphatase inhibitors and EDTA. Before SDS-PAGE, the total cellular protein (50 μg) were incubated with 50 units of calf intestinal alkaline phosphatase (ALP) in ALP buffer (100 mM NaCl, 50 mM Tris-HCl, 10 mM MgCl_2_, 1 mM dithiothreitol, pH 7.9) at 37 °C for 1 h.

### 4.5. RNA Isolation and a Semiquantitative Reverse-Transcription Polymerase Chain Reaction (RT-PCR)

Cells were collected by centrifugation, and total RNA was prepared by directly lysing cells in Trizol reagent (Invitrogen, Carlsbad, CA, USA) according to the manufacturer’s protocol. Two micrograms of total RNA was reverse-transcribed to synthesize complementary (c)DNA using the SuperScript III First-Strand Synthesis System for RT-PCR (Invitrogen). Two microliters of the first-strand reaction was used for the PCR amplification with specific primers for FasL or glyceraldehyde 3-phosphate dehydrogenase (GAPDH) primers, and it was carried out in the Gene Amp PCR system 2400 (Perkin-Elmer). Primers for the PCR analysis were as follows: FasL forward 5′-TCTCAGACGTTTTTCGGCTT-3′ and reverse 5′-AAGACAGTCCCCCTTGAGGT-3′; and GAPDH forward 5′-ACCACAGTCCATGCCATCAC-3′ and reverse 5′-TCCACCACCCTGTTGCTGTA-3′. The PCR parameters comprised a cycle of 5 min at 94 °C, 30 cycles of 50 s at 94 °C, 50 s at 58 °C, and 50 s at 72 °C, followed by a cycle of 5 min at 72 °C. PCR products were separated on 2% agarose gels and visualized with SYBR green.

### 4.6. Flow Cytometric Analysis

Briefly, cells were collected in phosphate-buffered saline (PBS) and resuspended in annexin-binding buffer (10 mM HEPES, 140 mM NaCl, and 2.5 mM CaCl_2_, pH 7.4). Cells were stained with 5 μL Annexin V-Alexa Fluor 488 and propidium iodide (PI, 1 μg/mL) at room temperature for 15 min in the dark. Apoptotic cell death was analyzed by flow cytometry using the Annexin V-conjugated Alexa Fluor488 Apoptosis Detection Kit according the manufacturer’s instructions (Molecular Probes, Eugene, OR, USA). Stained cells were analyzed by FACScan flow cytometry using CellQuest 3.3 analysis software (Becton Dickinson, San Jose, CA, USA) [33].

### 4.7. Antitumor Nude Mice Experiment

Male Balb/c AnN-Foxn 1 nu/CrlNarl nude mice (4~5 weeks old) were purchased from the National Laboratory Animal Center (Taipei, Taiwan) and kept in a specific pathogen-free environment within barrier systems. All animal experimental procedures were conducted and approved by the Institutional Animal Care and Use Committee of Taipei Medical University. At 24 and 72 h before tumor cell transplantation, mice were intraperitoneally (i.p.) administered with 35 mg/kg cyclophosphamide to improve the engraftment rates. HL-60 cells (10^7^ cells) were first mixed with an equal volume of Matrigel™ Basement Membrane Matrix (BD Biosciences Taiwan), and then nude mice were subcutaneously injected between the scapulae with HL-60/Matrigel. After transplantation for 10 days, mice received an i.p. injection of either 25 μL DMSO (vehicle), 12.5 mg/kg DHTS, or 25.0 mg/kg DHTS once a day for a week. At the end of the experiment, mice were sacrificed, and tumor specimens were excised, photographed, and weighed.

### 4.8. Statistical Analysis

Data are presented as the mean and stand deviation (SD) of the indicated number of independently performed experiments. Statistical analysis was done using a one-way analysis of variance (ANOVA) test, and differences were considered significant at *p* < 0.05.
